# Effect of Si and Holding Time on Ti_2_Al_20_La Phase in Al-Ti-La Intermediate Alloy

**DOI:** 10.3390/ma17133134

**Published:** 2024-06-26

**Authors:** Hu Da, Xudong Tian, Jiazhi An, Wanwu Ding, Jianchao Chen, Haicun Yu, Haixia Zhang

**Affiliations:** 1 Gansu Computing Center, Lanzhou 730050, China; 2 School of Materials Science and Engineering, Lanzhou University of Technology, Lanzhou 730050, China; 3 State Key Laboratory of Advanced Processing and Reuse of Non-Ferrous Metals, Lanzhou University of Technology, Lanzhou 730050, China

**Keywords:** Al-Ti-La alloy, Ti_2_Al_20_La, Si element, holding time

## Abstract

The effects of holding time and Si on the content, shape size and structure of Ti_2_Al_20_La phase in Al-Ti-La intermediate alloy were investigated by an X-ray diffractometer, scanning electron microscope and transmission electron microscope. The results show that the volume fraction and aspect ratio of Ti_2_Al_20_La phase in Al-Ti-La intermediate alloy decrease significantly, from 21% and 2.3 without Si addition to 4% and 2.0 with the addition of 2.3 wt.% Si at a holding time of 15 min at 750 °C, respectively. The Si element will attach to the Ti_2_Al_20_La phase and form La-Si binary phase at the grain boundary of α-Al. With the increase of holding time from 15 min to 60 min, the content of Ti_2_Al_20_La phase in the alloy gradually decreases and the size decreases significantly. Meanwhile, Al_11_La_3_ will dissolve and disappear, while the content of La-Si binary phase increases, and part of Ti_2_Al_20_La phase transforms into Ti_2_(Al_20−*x*_,Si*_x_*)La phase.

## 1. Introduction

Al-Si alloy is widely used in automotive, aerospace and other industries because of its excellent casting performance, high strength and easy processing [1,2,3,4,5]. However, the coarse α-Al phase and non-uniform distribution of needle sheet eutectic silicon in the as-cast condition of conventional Al-Si alloy will cause stress concentration, thereby significantly reducing the mechanical properties of the alloy. Grain refinement and eutectic Si modification are effective ways to improve the comprehensive mechanical properties of hypoeutectic Al-Si alloys. Research has shown that adding refiners and modifiers to aluminum melt is the most economical method, including Al-Ti-B [6], Al-Ti-C [7] and Al-B [8], and the metamorphic agents include Na [9], Sr [10] and RE [11], etc. In actual production, refiners and metamorphic agents are often added to aluminum melt in batches, which is not only complicated in process, but also unsatisfactory in the refining and metamorphic effect due to the “Si poisoning effect” [12].

In recent years, many scholars have developed novel intermediate alloys with both refining and modification effects [13,14,15,16]. Cui et al. [17] prepared a new Al-3B-5Sr intermediate alloy by in situ synthesis of aluminum melt. By adding 0.5 wt.% Al-3B-5Sr alloy to A356 alloy, the morphology of eutectic Si can be changed from needle/plate to fiber/ball. The size of α-Al grain is reduced from 1000 μm to 300 μm. Qiu et al. [18] indicated that adding 0.5 wt% Al-6Sr-7La intermediate alloy to A356 alloy, which is composed of α-Al, Al_4_Sr and Al_4_La phases, can reduce the secondary dendrite arm spacing of α-Al and transform sheet eutectic Si into fiber. The Al_4_La phase can be used as an effective hetero-nucleation substrate for α-Al and can exist stably at high temperatures, while the rare earth La reacts with Si to form La-Si intermetallic compounds. Zhao et al. [19] showed that adding 0.5% Al-Ti-C-Sr intermediate alloy to A356 alloy could reduce the secondary dendrite arm spacing of α-Al from 40 μm to 32.7 μm, and the eutectic Si changed from acicular/flake to fibrous/spherical. Wang et al. [20] found that Ti_2_Al_20_RE (RE = La/Ce) has a good lattice matching relationship with α-Al, which can promote the nucleation of α-Al in Al-7.0 Si-0.55Mg alloy. Quan et al. [21] showed that Ti_2_Al_20_RE (RE = Ce, La) has dynamic stability and excellent thermodynamic properties at high temperatures. Xu et al. [22] showed that Ti_2_Al_20_RE had higher hetero-nucleation ability than Al_3_Ti. However, the evolvement mechanism of these novel intermediate alloys after adding the Al-Si alloys, for example Ti_2_Al_20_RE (RE = Ce, La), is also not clear, especially how to avoid the “Si poisoning effect”.

The research group have prepared a new Al-3Ti-4.35La intermediate alloy rich in Ti_2_Al_20_La phase [23]. The preliminary results [23] show that the α-Al can be refined from 1116 μm to 105 μm by adding 0.2 wt.% Al-3Ti-4.35La to Al-7Si alloy. The morphology of eutectic silicon changes from coarse sheet to short rod and granular. It is concluded that Ti_2_Al_20_La plays an important role in refining and modifying eutectic Si, and the refining and modification effect of Al-Ti-La master alloy is obviously different due to the different shape, size and melt treatment process of Ti_2_Al_20_La. However, the relevant mechanism is still unclear. Based on this, this paper will deeply explore the microstructure evolution of Ti_2_Al_20_La phase in the Si alloy melt, which can provide a theoretical and experimental basis for the study of the refinement and metamorphism mechanism of Al-3Ti-4.35La intermediate alloy.

## 2. Experiment

Al-Ti-La intermediate alloy was prepared by in situ reaction of aluminum melt. First, Al powder (99.0 wt.%, 80–100 µm) and Ti powder (99.0 wt.%, 45–65 µm) were mixed at a molar ratio of Al:Ti = 3:1 and mechanically mixed at a speed of 170 r/min by a Pulaerisett-5 (Thermo Fisher Scientific, Waltham, MA, USA) high-speed planetary ball mill for 4 h. Then, the mixed powder was uniformly pressed into cylindrical preforms (density 50%, size: 25 × 50 mm) by WDW-100D universal test drawing machine (Jinan Testing Equipment Co., Ltd., Jinan, China). The preforms and lanthanum blocks were wrapped in aluminum foil and placed in DGG-9416A type electric thermostatic air drying oven (Dongguan Bell Experiment Equipment Co., Ltd., Dongguan, China) at 200 °C for 2 h for use. Proper amount of industrial pure aluminum (99.7 wt.%) was loaded into the clay crucible and heated in the silicon carbon rod resistance furnace to 1150 °C. Then, the precast and lanthanum blocks were pressed into the melt at the same time, stirred and kept warm for 4 min. After cooling to 860 °C, they were poured into the ring steel mold (size: inner diameter 45 mm, outer diameter 70 mm, height 70 mm) to obtain Al-Ti-La intermediate alloy.

**Figure 1:**
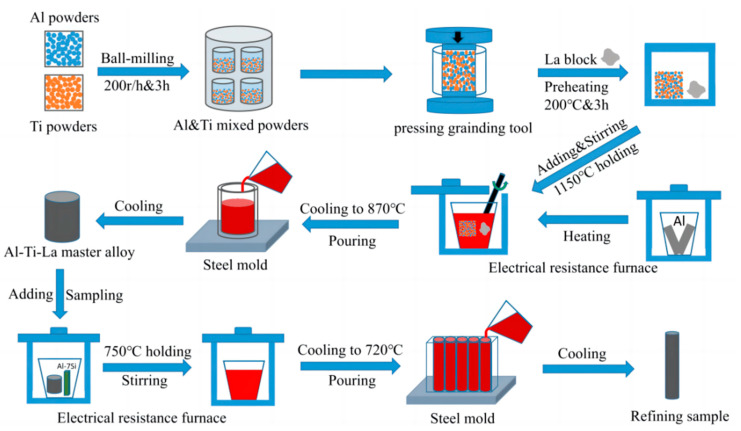
Experimental flow chart.

The Al-3Ti-4.35La intermediate alloy and Al-7Si alloy were put into a graphite crucible with a certain mass ratio and heated in a silicon carbon rod resistance furnace to melt at 750 °C. After fully mixing and mixing, the alloy was kept warm for 15 min, 30min and 60 min, respectively, and then 1 wt.% C_2_Cl_6_ was added to the melt for refining and degaging. After slagging, the obtained alloy melt was cast at 720 °C into a steel mold (outer diameter 140 mm, inner diameter 120 mm, outer height 210 mm) with a preheating temperature of 200 °C to prepare the Al-Ti-La-Si alloy sample. The experimental process is shown in Figure 1.

The chemical composition of Al-Ti-La alloy and Al-Ti-La-Si alloy was determined by X-ray fluorescence spectrometer (Panako Zetium, Breda, The Netherlands), and the results are given in Table 1. Al-Ti-La intermediate alloys and Al-Ti-La-Si alloys were characterized by Thermo ARL X-ray diffractometer (XRD, Thermo Fisher Scientific, Waltham, MA, USA) and monochromatic Cu Kα radiation at 2 θ angles ranging from 20 to 90°. The microstructure of the alloy was analyzed by Tescan Mira4 SEM and xplore 30 (Tescan, Brno, Czech Republic). The volume fraction and size of the second phase in the alloy were counted by Image Pro Plus 6.0, and 20 alloy topography images were analyzed. Talos F200X G2 transmission electron microscopy (TEM, Thermo Fisher Scientific, Waltham, MA, USA) with selected electron diffraction (SAED, Thermo Fisher Scientific, Waltham, MA, USA) was used to confirm the crystal structure, and samples for TEM observation were thinned by double-jet electrodeposition using a mixture of nitric acid.

## 3. Results and Discussion

### 3.1. Analysis of Al-Ti-La Intermediate Alloys

**Figure 2:**
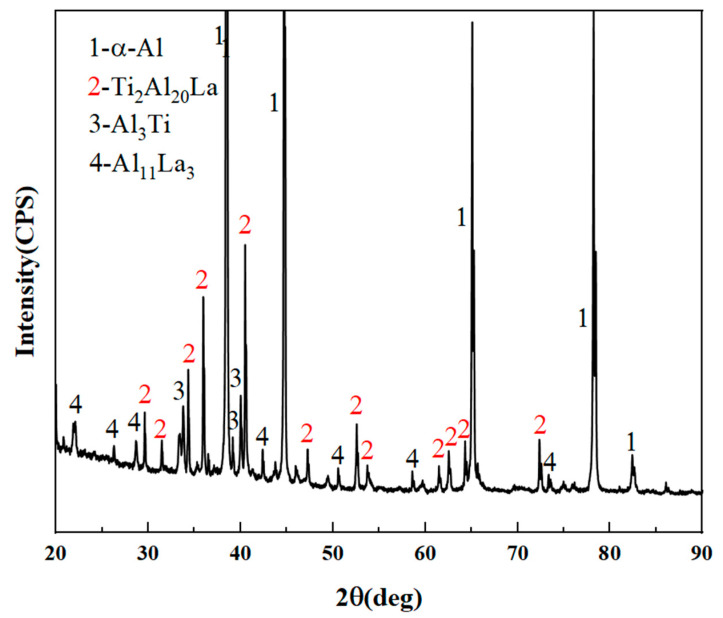
XRD pattern of Al-Ti-La intermediate alloy.

Figure 2 shows the XRD pattern of Al-Ti-La intermediate alloy. It can be seen from the figure that the main phases in the alloy are α-Al and Ti_2_Al_20_La phases, while a small amount of Al_3_Ti and Al_11_La_3_ phases exist. The author’s previous research results show that [23]: The content of Ti_2_Al_20_La in Al-Ti-La intermediate alloy has an important influence on its refining and metamorphic effect, and the higher the content of Ti_2_Al_20_La in the intermediate alloy, the better the refining and metamorphic effect. According to the intensity of the diffraction peak of Ti_2_Al_20_La, it is preliminatively determined that the intermediate alloy prepared in this paper is rich in Ti_2_Al_20_La.

**Figure 3:**
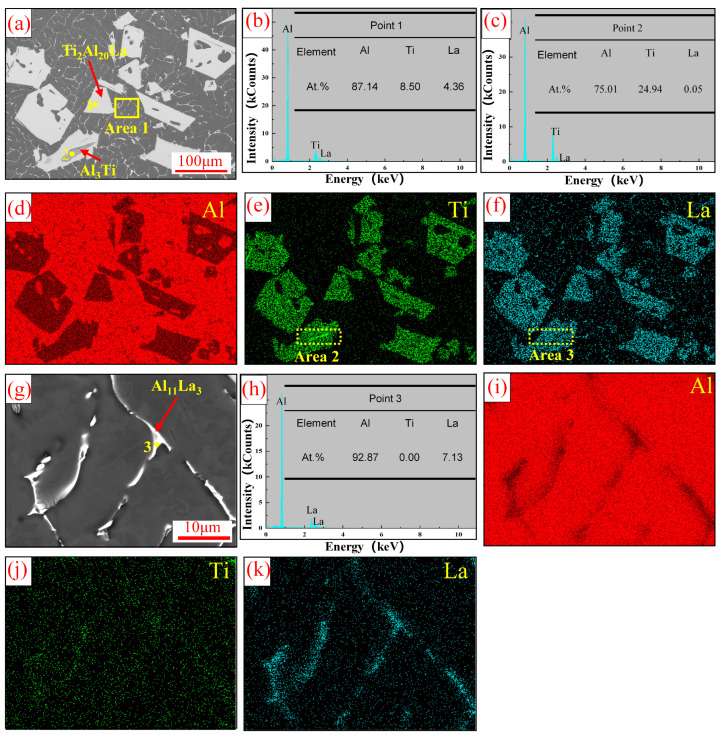
SEM and EDS results of Al-Ti-La intermediate alloy: (**a**) SEM; (**b**,**c**) are EDS point analysis results at Points 1 and 2 in (**a**); (**d**–**f**) shows the face scanning distribution of Al, Ti and La elements in (**a**); (**g**) The enlarged area of Area 1 in (**a**); (**h**) The EDS point analysis results at Point 3 in (**g**) are shown. (**i**–**k**) is the mapping of Al, Ti and La elements in (**g**).

Figure 3 shows the SEM and EDS analysis results of Al-Ti-La intermediate alloy. As can be seen from Figure 3a, a large number of white blocky structures are distributed in the alloy matrix, some of the white blocky structures are wrapped with striped gray phases and irregular fibrous white structures are uniformly distributed in the matrix. Figure 3b–k shows EDS analysis of three different tissues. It can be seen from the figure that Al, Ti and La elements are evenly distributed in the white massive tissue, and EDS analysis results at Point 1 indicate that the ratio of Al:Ti:La elements is close to 20:2:1. The gray phase wrapped with strips in the white massive structure is enriched with Ti element, and the EDS analysis shows that the ratio of Al:Ti element is close to 3:1. In the fibrous white structure, only Al and La elements are distributed, and the EDS point analysis shows that the element ratio of Al:La in this phase is close to 11:3. Combined with the XRD analysis results in Figure 2, the white bulk structure, gray phase and fibrous white structure in Al-Ti-La intermediate alloy are Ti_2_Al_20_La, Al_3_Ti and Al_11_La_3_ phases, respectively.

**Figure 4:**
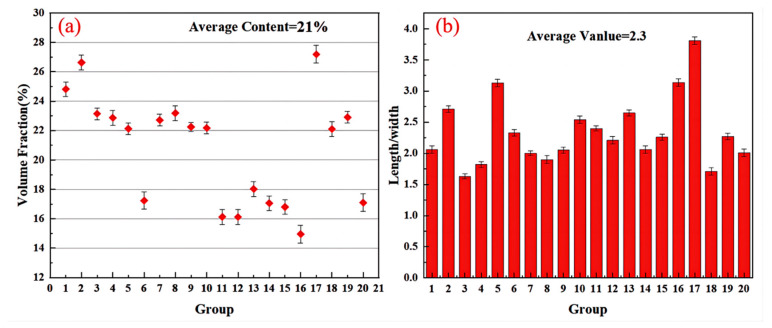
Volume fraction and size distribution of Ti_2_Al_20_La phase in Al-Ti-La alloy: (**a**) Ti_2_Al_20_La phase volume fraction diagram; (**b**) Ti_2_Al_20_La phase width ratio layout.

Figure 4 shows the statistical distribution of the volume fraction and aspect ratio of Ti_2_Al_20_La phase in Al-Ti-La intermediate alloy. It can be seen that the volume fraction of Ti_2_Al_20_La phase in Al-Ti-La intermediate alloy is 21%, the size distribution of Ti_2_Al_20_La phase is relatively uniform and the aspect ratio is about 2.3. Previous studies have shown [23] that when the relative content of Ti_2_Al_20_La in the intermediate alloy is high and the form is massive, the refining and metamorphism effect of Al-7Si alloy is the best. Therefore, the effect of Si element on the evolution behavior and stability of Ti_2_Al_20_La phase in melt can be effectively observed by adding Si element to this intermediate alloy for study.

### 3.2. Analysis of Al-Ti-La-Si Alloy under Different Holding Time

**Figure 5:**
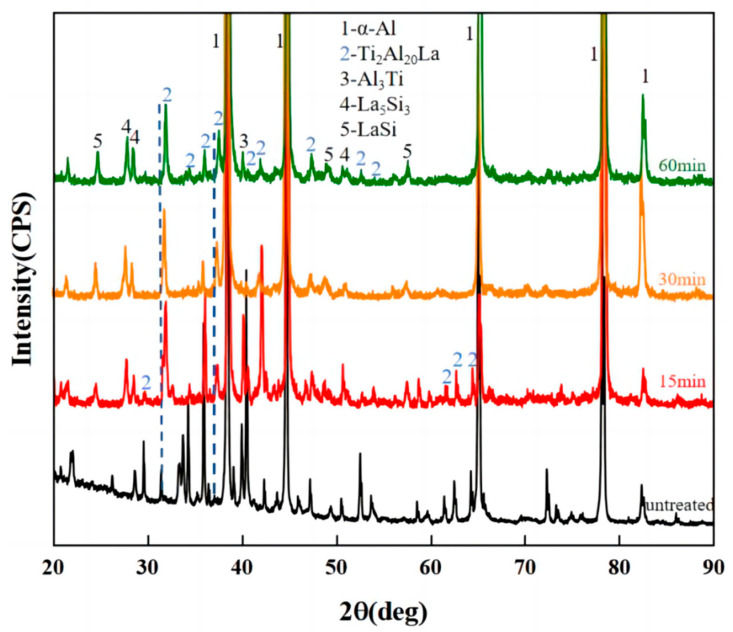
XRD pattern of Al-Ti-La-Si alloy at different holding times.

Figure 5 shows the XRD pattern of Al-Ti-La-Si alloy at different holding times. It can be seen that the main phases of Al-Ti-La intermediate alloy are still α-Al, Ti_2_Al_20_La and Al_3_Ti phases after Si element is added and the holding time is different, but the peak strength corresponding to Ti_2_Al_20_La significantly decreases with the extension of the holding time, and some peaks disappear. At the same time, the peak corresponding to the Al_11_La_3_ phase disappeared, and the La_5_Si_3_ phase and LaSi phase appeared. After extending the holding time, the Ti_2_Al_20_La phase content decreased, which may be due to the dissolution of a large amount of Ti_2_Al_20_La, which produced free [La], so that [La] and [Si] had more time to diffuse, so that more La-Si binary phases were formed. In addition, compared with Ti_2_Al_20_La on the standard PDF card, the diffraction peak of Ti_2_Al_20_La phase is found at the dotted line in Figure 5, and the offset distance gradually increases with the extension of holding time. It has been shown [24] that the Si element in Al-Si alloy will replace some Al atoms in Al compounds to form (Al, Si) intercompounds. Therefore, this study suggests that the peaks at 31.89° and 37.52° angles in Figure 5 may be Ti_2_(Al_20−*x*_,Si*_x_*)La formed by partial substitution of Al atoms in the Ti_2_Al_20_La phase by Si atoms.

**Figure 6:**
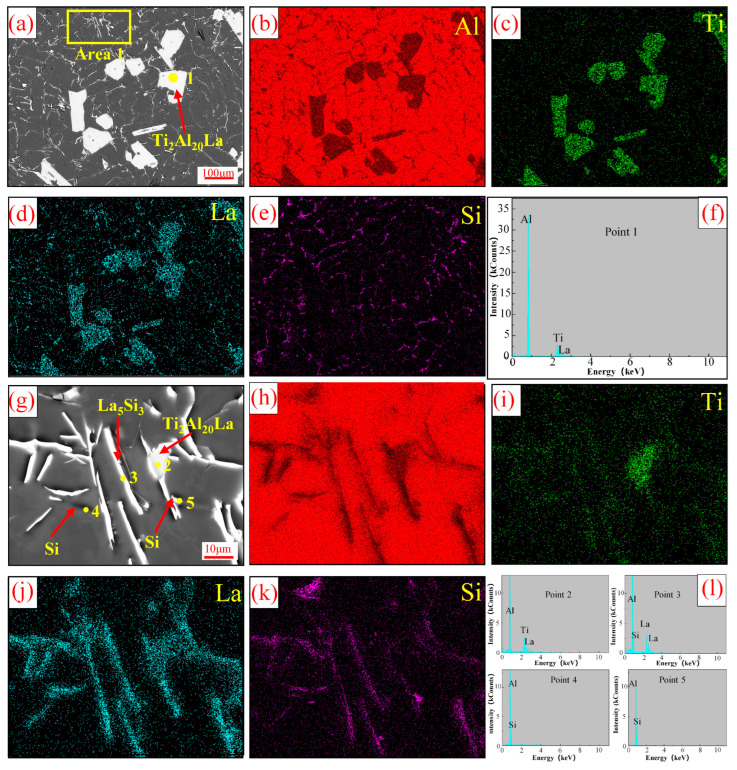
SEM and EDS results of Al-Ti-La-Si alloy held for 15 min: (**a**) SEM; (**b**–**e**) face scan distribution of Al, Ti, La and Si elements in (**a**); (**f**) EDS point analysis results at Point 1 in (**a**); (**g**) the enlarged area of Area 1 (**a**); (**h**–**k**) mapping of Al, Ti, La and Si elements in (**g**); (**l**) EDS point analysis spectra of Point 2, Point 3, Point 4 and Point 5 in (**g**).

Figure 6 shows the SEM and EDS results analysis of Al-Ti-La-Si alloy held for 15 min. It can be seen from Figure 6a that white massive structures are distributed in the alloy matrix, while irregular rod-like white structures and gray structures are distributed at the grain boundaries. Combined with the EDS quantitative analysis of different tissues as shown in Figure 6b–f and Table 2, and XRD analysis as shown in Figure 5, it can be determined that the white massive tissue is Ti_2_Al_20_La phase. Figure 6g is a magnified image of the fibrous structure in region 1 of Figure 6a. It can be seen from the figure that the fibrous structure is mainly composed of white rod-like and granular structures, while some small gray rod-like structures are distributed in the matrix. Combined with EDS analysis in Figure 6h–l and XRD analysis in Figure 5, the white rod-like structure is judged to be La_5_Si_3_ phase. Considering that the existing form of Si in Al-Si alloy is mainly eutectic Si, which does not produce Al-Si binary phase 25, the white granular structure and gray rod-like structure in Figure 6g are eutectic Si.

**Figure 7:**
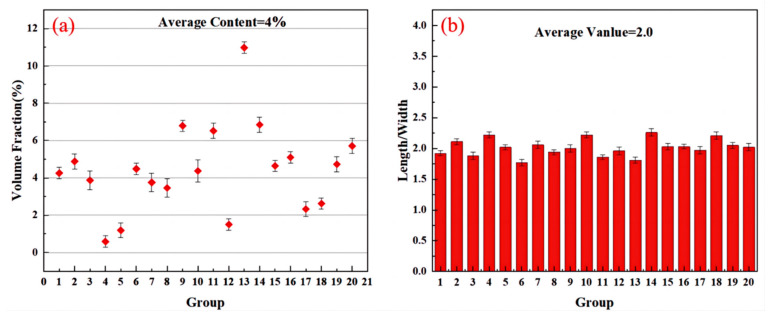
Volume fraction and size distribution of Ti_2_Al_20_La in Al-Ti-La-Si held for 15 min: (**a**) second phase volume fraction diagram; (**b**) second phase length and width ratio of the layout.

Figure 7a shows the volume fraction of Ti_2_Al_20_La phase in Al-Ti-La-Si alloy held for 15 min, and its value is 4%, which is significantly lower than 21% before Si element is added. In addition, it can be seen from Figure 7b that the average aspect ratio of Ti_2_Al_20_La phase is 2.0, which is also lower than 2.3 when Si element is not added.

**Figure 8:**
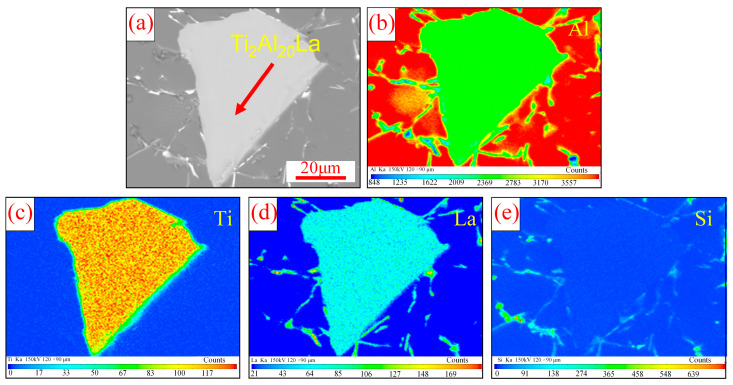
EPMA analysis of Ti_2_Al_20_La in Al-Ti-La-Si alloy held for 15 min: (**a**) SEM image of Ti_2_Al_20_La; (**b**–**e**) mapping of Al, Ti, La and Si elements in Figure 8.

In order to determine the distribution of elements after dissolution of Ti_2_Al_20_La, EPMA analysis was carried out, as shown in Figure 8. As can be seen from Figure 8a, after 15 min of heat preservation, the sharp edges and corners of the original block of Ti_2_Al_20_La become more rounded, indicating that Ti_2_Al_20_La has dissolved from its edge, and there has been segregation of Ti element at the edge of Ti_2_Al_20_La (as shown in Figure 8c). And part of the free [Ti] begins to diffuse around. In addition, it can be seen from Figure 8d,e that the distribution of rare earth elements La and Si has a strong correlation, which indicates that [La] produced by dissolution mainly combines with Si to form La-Si intermetallic compounds, while excessive Si elements precipitate at the grain boundaries in the form of eutectic Si.

**Figure 9:**
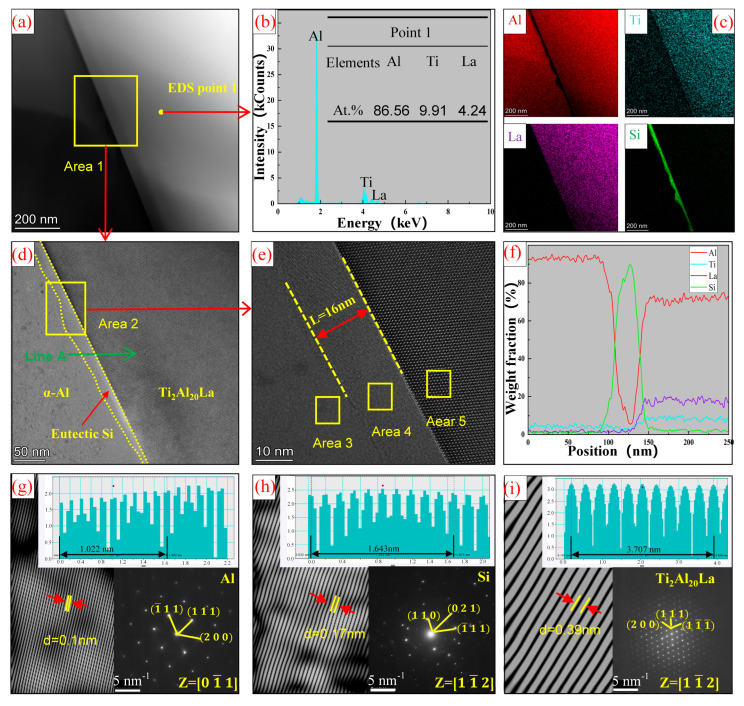
(**a**) Bright field image of Ti_2_Al_20_La phase in Al-Ti-La-Si alloy held for 15 min; (**b**) EDS point analysis energy spectrum at Point 1 in (**a**); (**c**) mapping of Al, Ti, La and Si elements in (**a**); (**d**) enlarged Area 1 of the interface between Ti_2_Al_20_La phase and eutectic Si in (**a**); (**e**) High-resolution HAADF diagram of Area 2 in (**d**); (**f**) scan results of Line A in (**d**); (**g**–**i**) are the inverse Fourier transform graphs in Area 3, Area 4 and Area 5 in (**e**), respectively.

Figure 9 shows the TEM analysis of the interface between Ti_2_Al_20_La phase and eutectic Si in Al-Ti-La-Si alloy held for 15 min. Combined with EDS analysis Figure 9a–c,f, it is obvious that there is an enrichment region of Si elements between α-Al and Ti_2_Al_20_La. The phase relationship between Si and Ti_2_Al_20_La was further analyzed. High-resolution analysis was performed on region 2 in Figure 9d, as shown in Figure 9e. The analysis showed that the width of the rich region was about 16 nm. Region 3, region 4 and region 5 corresponding to α-Al, eutectic Si and Ti_2_Al_20_La were analyzed by inverse Fourier transform to analyze their lattice spacing and phase relationships, as shown in Figure 9g–i. The analysis shows that: The lattice spacing of α-Al, eutectic Si and Ti_2_Al_20_La is 0.1 nm, 0.17 nm and 0.39 nm, respectively, and the lattice is arranged neatly, and there is no obvious atomic distortion region, that is, the eutectic Si is only attached to the Ti_2_Al_20_La phase, but it will not have a significant impact on the structure of Ti_2_Al_20_La phase.

**Figure 10:**
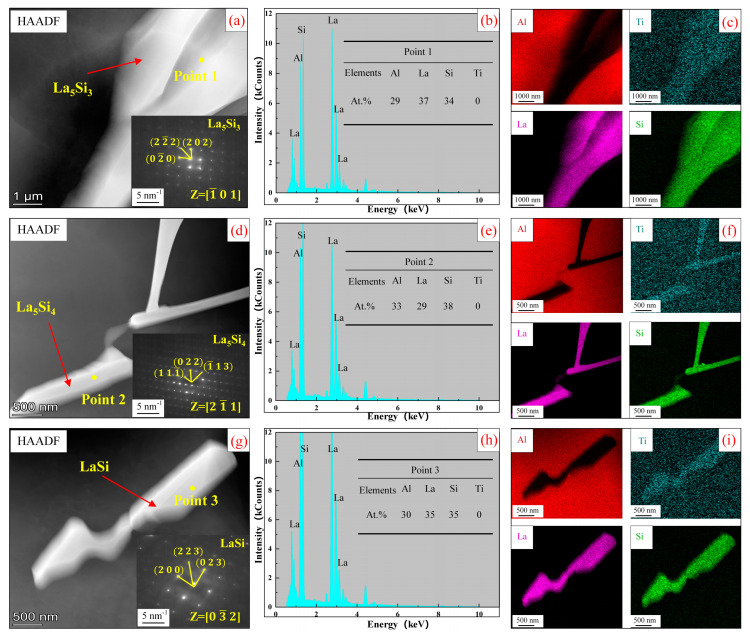
(**a**,**d**,**g**) are HAADF images of other rare earth phases in Al-Ti-La-Si alloy held for 15 min. (**b**,**e**,**h**) EDS point analysis energy spectra of Point 1, Point 2 and Point 3 in (**a**,**d**,**g**); (**c**,**f**,**i**) mapping of Al, Ti, La and Si elements in (**a**,**d**,**g**).

Figure 10 shows TEM and EDS analysis of other phases in Al-Ti-La-Si alloy held for 15 min. It can be seen that the white phases in Figure 10a,d,e are La_5_Si_3_ phase, La_5_Si_4_ phase and LaSi phase, respectively, and the corresponding SAED analysis further confirms the existence of these phases.

From the above analysis, it can be seen that after Si element is added to Al-Ti-La alloy, new rare earth phases La_5_Si_3_, La_5_Si_4_ and LaSi are formed, among which La_5_Si_4_ phase is not found in XRD and SEM analysis of Al-Ti-La-Si alloy. In order to analyze this reason, the formation energies of rare earth phases La_5_Si_3_, La_5_Si_4_ and LaSi were calculated. The formation energies of La_5_Si_3_, La_5_Si_4_ and LaSi were −0.679 eV, −0.772 eV and 0.001 eV, respectively, indicating that La_5_Si_3_ and LaSi could exist stably. La_5_Si_4_ is not stable. Studies have shown [25] that the space group where La_5_Si_4_ phase resides is P41212, which is in metastable state and tends to decompose into La_5_Si_3_ and LaSi. Therefore, La_5_Si_3_ and LaSi phases are the main phases that can exist stably in large quantities at room temperature.

**Figure 11:**
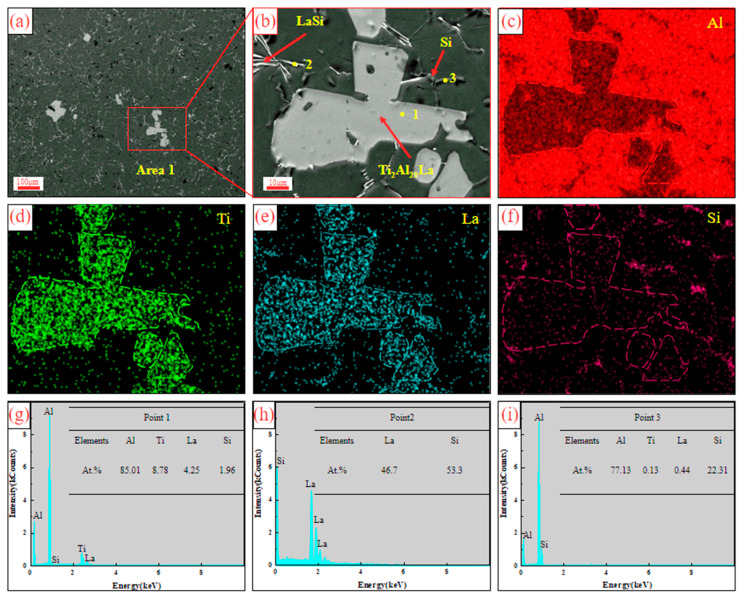
SEM and EDS results of Al-Ti-La-Si alloy held for 30 min: (**a**) SEM; (**b**) enlarged view of Area 1 in (**a**); (**c**–**f**) is the mapping of Al, Ti, La and Si elements in (**b**); (**g**–**i**) is the EDS point analysis energy spectrum of Point 1, Point 2 and Point 3 in (**b**).

Figure 11 shows the SEM and EDS analysis of Al-Ti-La-Si alloy held for 30 min. It can be seen from the figure that the phase shape of Ti_2_Al_20_La becomes more rounded and smaller. The region 1 in Figure 11a is enlarged as shown in Figure 11b. Combined with the surface scanning results (Figure 11c–f), it is shown that some Si elements are distributed around the Ti_2_Al_20_La phase. In particular, it can be seen from the EDS scanning results of Point 1, Figure 11g, that there is a small amount of Si elements in the Ti_2_Al_20_La phase. And the atomic ratio of (Al, Si): Ti: La is close to 20:2:1, which is mainly due to the similar atomic radius of Al and Si atoms. Si atoms will replace part of Al atoms in Ti_2_Al_20_La, resulting in a decrease in the proportion of Al atoms [26]. A small number of Si atoms (1.96 At.%) exist in the matrix of Ti_2_Al_20_La, forming the Ti_2_(Al_20−*x*_,Si*_x_*)La phase with the same structure as Ti_2_Al_20_La. As shown in Figure 11h,i of EDS point scan results at Points 2 and 3, the white rod-like structure is La-Si binary phase and the gray structure is eutectic Si.

**Figure 12:**
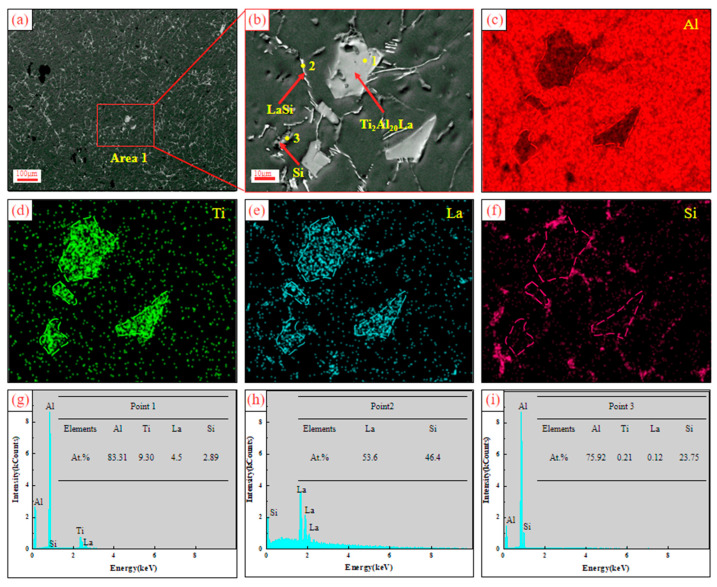
SEM and EDS results of Al-Ti-La-Si alloy held for 60 min: (**a**) SEM; (**b**) enlarged view of Area 1 in (**a**); (**c**–**f**) mapping of Al, Ti, La and Si elements in (**b**); (**g**–**i**) EDS point analysis energy spectra of Point 1, Point 2 and Point 3 in (**b**).

Figure 12 shows the SEM and EDS analysis of Al-Ti-La-Si alloy held for 60 min. It can be seen from the figure that the size of Ti_2_Al_20_La phase after holding for 60 min is smaller than that after holding for 30 min. The region 1 in Figure 12a was enlarged, as shown in Figure 12b, and the element surface scanning analysis of Figure 12b showed that the aggregation distribution of Si elements was more obvious around the Ti_2_Al_20_La phase, and the EDS point scanning results at the junction Point 1 showed that the ratio of Si elements in the Ti_2_Al_20_La phase increases to 2.89At. %, but the ratio of (Al, Si):Ti:La atoms is still close to 20:2:1, indicating that the trend of Si atoms replacing Al atoms is more obvious. As shown in Figure 12h,i of EDS point scan results at Points 2 and 3, the white rod-like structure is La-Si binary phase and the gray structure is eutectic Si.

**Figure 13:**
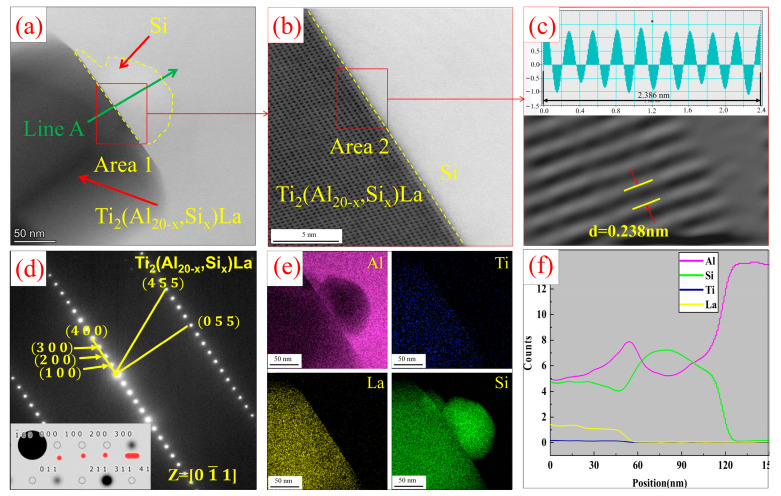
TEM analysis of Al-Ti-La-Si alloy held for 60 min: (**a**) TEM bright field images of Ti_2_(Al_20−*x*_,Si*_x_*)La and Si; (**b**) high-resolution map of Area 1 in (**a**); (**c**) Fourier transform diagram of Area 2 in (**b**); (**d**) diffraction spots of Ti_2_(Al_20−*x*_,Si*_x_*)La La; (**e**) mapping of Al, Ti, La and Si elements in (**a**); (**f**) EDS line scan results of Line A in (**a**).

Figure 13 shows the interfacial relationship analysis between Ti_2_(Al_20−*x*_,Si*_x_*)La phase and eutectic Si in Al-Ti-La-Si alloy held for 60 min. Combined with EDS surface scanning and line scanning, it is found that Si elements exist at the boundary and inside of rare earth phase Ti_2_Al_20_La. In order to further analyze the orientation relationship between Si and Ti_2_(Al_20−*x*_,Si*_x_*)La phase, high-resolution analysis, inverse Fourier transform analysis and diffraction spot calibration of the interface were carried out, as shown in Figure 13b–d. The results show that the atoms at the interface of Si and Ti_2_(Al_20−*x*_,Si*_x_*)La phase are arranged neatly without obvious lattice distortion. Due to the existence of atomic distortion in the process of Si replacing Al atom, the internal lattice fringe of Ti_2_(Al_20−*x*_,Si*_x_*)La is locally deformed and distorted [27]. Ti_2_(Al_20−*x*_,Si*_x_*)La and Ti_2_Al_20_La belong to the same face-centered cubic structure. The comparison between the theoretical standard diffraction spots of Ti_2_Al_20_La and the actual diffraction spots of Ti_2_(Al_20−*x*_,Si*_x_*)La is shown in the illustration in Figure 13d. The black dots in the figure represent the crystal face existing in the theoretical Ti_2_Al_20_La. The black circles are diffraction spots resulting from extinction on a particular crystal plane, and the red dots are the actual diffraction spots of Ti_2_(Al_20−*x*_,Si*_x_*)La. Due to the action of Si, superlattice diffraction spots reappear at (100), (200), (300) and (400) crystal planes, indicating that the Ti_2_(Al_20−*x*_,Si*_x_*)La has a long-period structure. Due to the transition of the crystal structure of Ti_2_(Al_20−*x*_,Si*_x_*)La from disorder to order [28], its period is four times that of Ti_2_Al_20_La and its lattice structure is similar to that of Ti_2_Al_20_La. Ti_2_(Al_20−*x*_,Si*_x_*)La crystal face spacing d = 0.238 nm is lower than Ti_2_Al_20_La’s d = 0.39 nm. This shows that Si does not react directly with Ti_2_Al_20_La to form the Ti_2_(Al_20−*x*_,Si*_x_*)La phase, but forms the Ti_2_(Al_20−*x*_,Si*_x_*)La phase by diffusing into the lattice of Ti_2_Al_20_La to replace Al atoms in certain positions.

**Figure 14:**
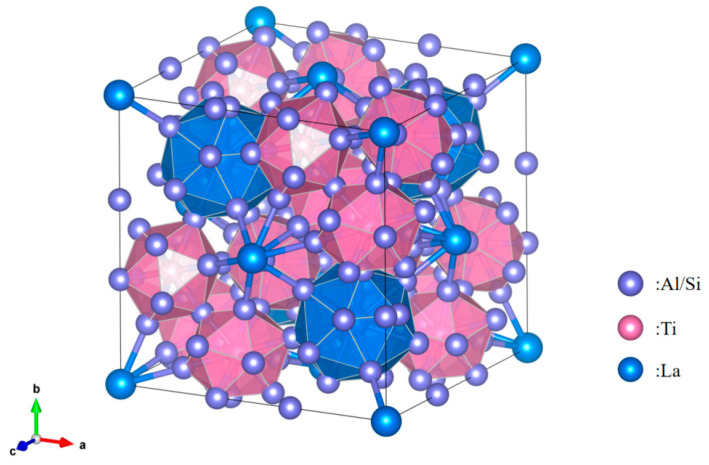
Atomic structure model of Ti2(Al_20−*x*_,Si*_x_*)La.

Crystalmaker 11 software was used to draw the atomic structure model of Ti_2_(Al_20−*x*_,Si*_x_*)La, as shown in Figure 14. The atomic radii of Al, Si, Ti and La were 1.25 nm, 1.11 nm, 1.9 nm and 1.95 nm, respectively [29], in which the atomic radii of Al and Si were similar. The atomic radius of Si, Ti and La atoms differs greatly. Therefore, Si will replace the positions of some low-energy Al atoms in Ti_2_Al_20_La, forming Ti_2_(Al_20−*x*_,Si*_x_*)La phase, which has a similar structure to Ti_2_Al_20_La. Due to the small radius of Si atom, the lattice spacing of Ti_2_(Al_20−*x*_,Si*_x_*)La phase after replacement is smaller than Ti_2_Al_20_La.

## 4. Conclusions

The content, shape size and structure of Ti_2_Al_20_La phase in Al-Ti-La intermediate alloy with Si addition under different holding times were studied. The main conclusions of this paper are as follows:

(1) The aspect ratio of Ti_2_Al_20_La phase in Al-3Ti-4.35 intermediate alloy with a holding time of 15 min at 750 °C is 2.0 (with 2.3 wt% Si addition), which has the efficient refining and modification effects, although the volume fraction of Ti_2_Al_20_La phase decreased significantly from 21% (without Si addition) to 4% (with 2.3 wt% Si addition), respectively.
(2) The Si in the Al-Ti-La intermediate alloy will attach to the Ti_2_Al_20_La phase, which is the main reason for the avoidance of the “Si poisoning effect” with the addition of Al-Ti-La alloy to Al-7Si alloy. Meanwhile, some new rare earth phases La_5_Si_3_, La_5_Si_4_ and LaSi will form and distribute in the grain boundary of α-Al.
(3) With the increase of holding time from 15 min to 60 min, Si will replace the positions of some low-energy Al atoms in Ti_2_Al_20_La, forming Ti_2_(Al_20−*x*_,Si*_x_*)La phase, which has a similar structure to Ti_2_Al_20_La and has refining and modification effects.

## Figures and Tables

**Table 1:** Composition of Al-Ti-La and Al-Ti-La-Si (%).

Elements	Ti	La	Si	Cl	Zn	Fe	Al
Al-Ti-La	2.883	4.277	0.019	0.038	0.035	0.037	Bal.
Al-Ti-La-Si	1.874	2.875	2.301	0.072	0.039	0.095	Bal.

**Table 2:** Element content of each point in Figure 6.

Point	Elements At.%			
	Al	Ti	La	Si
1	84.87	8.81	4.58	1.74
2	85.29	8.21	4.68	1.82
3	81.44	0.00	8.94	9.62
4	83.55	0.00	0.80	15.66
5	62.37	0.05	0.96	36.62

## Data Availability

The original contributions presented in the study are included in the article; further inquiries can be directed to the corresponding author.
